# Colour Test Chart

**Published:** 1892-03

**Authors:** 


*Colour Test Chart.* By JOHN BROWN, M.D. London: Burroughs, Wellcome & Co.

This test for colour-blindness is based upon the theory of Edridge Green, and will be found of use in examination of the colour-perception. But where a question as to defect in colour-vision becomes important, most experts would prefer Holmgren's wools.

